# Very severe oligozoospermia with AZFc microdeletion patients may affect intracytoplasmic sperm injection clinical outcomes: A propensity score matching analysis

**DOI:** 10.1002/rmb2.12596

**Published:** 2024-07-09

**Authors:** Huan Zhang, Huanzhu Li, Shujuan Ma, Shuoping Zhang, Wen Li, Yifan Gu, Erchen Zhang, Liang Hu

**Affiliations:** ^1^ NHC Key Laboratory of Human Stem Cell and Reproductive Engineering, School of Basic Medical Sciences Central South University Changsha Hunan China; ^2^ School of Medicine Hunan Normal University Changsha Hunan China; ^3^ Clinical Research Center for Reproduction and Genetics in Hunan Province Reproductive and Genetic Hospital of CITIC‐Xiangya Changsha Hunan China; ^4^ National Engineering and Research Center of Human Stem Cells Changsha Hunan China

**Keywords:** azoospermia factor, intracytoplasmic sperm injection, propensity score matching, severe oligozoospermia, Y chromosome microdeletion

## Abstract

**Purpose:**

To explore whether spermatozoa from AZFc microdeletion patients affect their outcomes of intracytoplasmic sperm injection (ICSI).

**Methods:**

Eighty‐five patients with AZFc microdeletion were recruited. A control group of one hundred and forty patients with severe oligozoospermia but without AZF microdeletion was selected using propensity score matching analysis with a 1:2 nearest neighbor algorithm ratio. The ICSI outcomes of the two groups were compared.

**Results:**

AZFc microdeletion had lower rates of normal fertilization (73% vs. 80%, *p* = 0.17) and high‐quality embryos (44% vs. 58%, *p* = 0.07) than the control group. There was no significant difference in the clinical pregnancy rate, miscarriage rate, and live birth rate between the two groups. When the sperm concentration was <1 million/mL, the AZFc microdeletion group exhibited lower rates of fertilization (71% vs. 80%, *p* = 0.03), high‐quality embryo (44% vs. 58%, *p* = 0.02), clinical pregnancy (57% vs. 76%, *p* = 0.02), and live birth (49% vs. 72%, *p* = 0.01) than the control group. However, if sperm concentration was ≥1 million/mL, no significant differences were found.

**Conclusion:**

If the sperm concentration is <1 million/mL, AZFc microdeletion do have a detrimental effect on most outcomes of ICSI.

## Abstract 2

We investigated the outcomes of ICSI treatment for those with AZFc deletions by using propensity score matching. Our results showed that in the subgroup of those with extremely low concentrations of spermatozoa, AZFc deletions not only decreased the fertilization rate and high‐quality embryo rate but also the clinical pregnancy rate and live birth rate.

## 1 INTRODUCTION

Couples who cannot become pregnant after a year of unprotected sexual intercourse are diagnosed with infertility. Male factor infertility is responsible for approximately half of all infertility cases.^1^ The primary indicators of male infertility are abnormal sperm parameters, which can be caused by a range of factors such as genetic factors, urogenital tract infections, varicocele, environmental conditions, and lifestyle choices. Y chromosome microdeletion is a common genetic cause of male infertility, with a prevalence of 2%–5% and 5%–10% in severe oligozoospermia and azoospermia, respectively.^2^, ^3^ Both the European Association of Urology (EAU) and the American Society for Reproductive Medicine (ASRM) have issued guidelines, recommending that men with severe oligozoospermia (sperm concentration below 5 million/mL) and nonobstructive azoospermia undergo Y chromosome microdeletion testing.^3^, ^4^

It is widely recognized that a segment located on the long arm of the Y chromosome, known as the azoospermia factor (AZF), governs the process of spermatogenesis. The AZF can be subdivided into three separate, nonoverlapping regions, namely, the AZFa, AZFb, and AZFc regions.^5^, ^6^ Due to the abundance of highly similar sequences (amplicons) and palindrome sequences, the AZFc region is susceptible to structural rearrangement. Microdeletion of the AZFc region accounts for 57% of all AZF microdeletion, typically occurring through nonhomologous recombination.^7^ The complete absence of the AZFa and AZFb regions is related to azoospermia and sterility, with an anticipated failure certainty regarding sperm harvesting through testicular microdissection (mTESE).^4^ On the other hand, microdeletion of the AZFc region manifests as various pathological features, with 46% exhibiting Sertoli cell‐only syndrome, 38.2% exhibiting maturation arrest, and 15.7% exhibiting hypospermatogenesis.^7^ A meta‐analysis of thirty‐two studies revealed that the average success rate of extracting spermatozoa through microsurgery is 47%.^7^ In addition, AZFc microdeletion can cause severe oligozoospermia and cryptozoospermia.^3^, ^4^ Diagnosis of cryptozoospermia is established if no spermatozoa are observed in the wet preparations and rare spermatozoa are observed in the pellet obtained by centrifugation of the semen at 3000 × g for 15 min.^8^

Patients with AZFc microdeletion have limited spermatozoa in their semen or post‐mTESE, thus requiring the use of ICSI to achieve a successful pregnancy. Some studies indicate that individuals with AZFc microdeletion may experience a decrease in fertilization rates following ICSI.^9^, ^10^ A meta‐analysis of twelve studies indicated that ICSI‐assisted pregnancy with spermatozoa from AZFc microdeletion patients does not lead to a reduction in the quality of embryos, clinical pregnancy rates, miscarriage rates, or live birth rates.^11^ However, a retrospective study of three hundred and forty‐five ICSI cycles conducted on two hundred and ninety‐three AZFc microdeletion patients with azoospermia or severe oligozoospermia revealed that the implantation rate, number of transferable embryo cycles, clinical pregnancy rate, and live birth rate of the AZFc microdeletion group were all lower than those of the control group.^12^ It is important to note that previous studies have not fully considered the impact of female factors on the clinical outcomes of ICSI, nor have they factored in the possibility that distinct sperm sources and concentrations of AZFc microdeletion patients may affect the findings.

To reduce the impact of female factors on ICSI outcomes, this study employed propensity score matching to retrospectively examine the laboratory and clinical pregnancy results of AZFc microdeletion patients. Additionally, stratified analysis of sperm concentration was conducted to assess the impact of sperm concentration on the clinical outcomes of ICSI for patients with AZFc microdeletion.

## 2 MATERIALS AND METHODS

### 2.1 Study population

**FIGURE 1:**
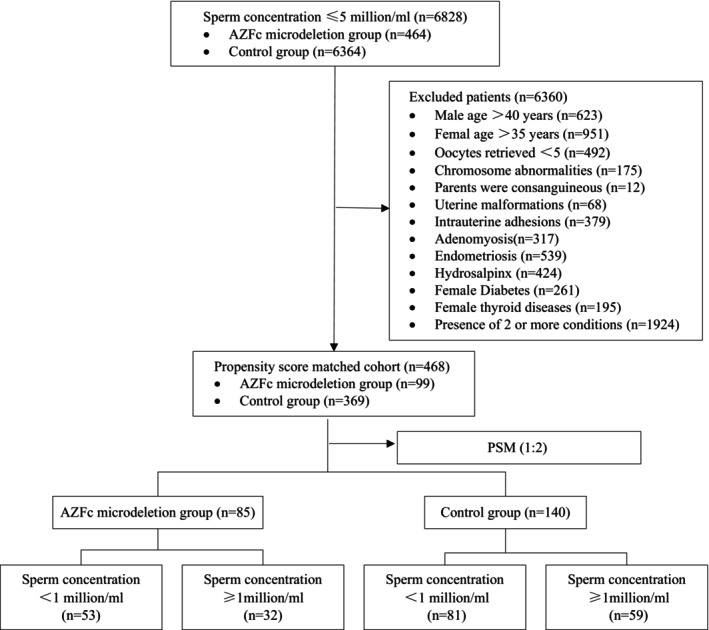
The study flowchart. PSM, propensity score matching.

The study population was recruited from patients undergoing assisted reproduction at the Reproductive and Genetic Hospital of CITIC‐Xiangya from January 2017 to December 2021 in China. A total of six thousand eight hundred and twenty‐eight male patients with infertility who had a sperm concentration of less than 5 million/mL (from cryptozoospermia to 3.7 million/mL) on two separate occasions underwent a comprehensive evaluation, including a physical examination, karyotype analysis, and AZF examination. Patients who had an abnormal karyotype, a consanguineous family history, severe varicocele, over 40 years of age, and whose female partners were over 35 years of age and had fewer than five oocytes retrieved, uterine malformations, moderate‐to‐severe intrauterine adhesions, adenomyosis, endometriosis, hydrosalpinx, diabetes, or thyroid diseases were excluded. A 1:2 propensity score was used to match the age and sperm concentration of the male, as well as the age, the number of retrieved oocytes, the protocols of controlled ovarian stimulation, and the anti‐Müllerian hormone (AMH) of the female partner. Finally, the research cohort comprised eighty‐five patients with AZFc microdeletion, whereas the control group comprised one hundred and forty patients (Figure 1). The primary outcomes evaluated were the number of retrieved oocytes, the number of MII oocytes, the normal fertilization rate, and the rate of high‐quality embryos on Day 3 in the first cycle of oocyte retrieval. Additionally, the clinical pregnancy rate, miscarriage rate, and live birth rate of the first transfer cycle were also assessed.

### 2.2 Ethical approval

The study was approved by the Institutional Review Board (IRB) of the Reproductive and Genetic Hospital of CITIC‐Xiangya (LL‐SC‐2022‐26). Our study was conducted in compliance with the principles of the Helsinki Declaration and was in alignment with medical ethics.

### 2.3 Y chromosome microdeletion testing

The Y chromosome microdeletion tests were performed in the genetic laboratory of the Reproductive and Genetic Hospital of CITIC‐Xiangya. DNA extraction from peripheral blood was performed in accordance with the company's instructions (Promega). The extracted DNA was then stored at −20°C. According to the recommendation of EAA/EMQN,^13^ six sequence tag sites (STSs) should be utilized for multiplex PCR to detect AZF microdeletion. The six STSs were in three regions of the AZFa region (sY84, sY86), the AZFb region (sY127, sY134), and the AZFc region (sY254, sY255). According to the company's instructions (Tellgen, Shanghai, China), two tubes of multiplex PCR amplification and four channels of fluorescence detection were performed. Additionally, the male sex determination gene (SRY) and a zinc finger protein coding gene (ZFY) were set as internal controls.

### 2.4 ART treatments

Controlled ovarian stimulation protocols included agonists protocol and antagonists protocol, which were determined based on patients' basal characteristics and performed as previously published.^14^ Patients suffering from severe oligozoospermia were required to abstain from ejaculation for 2–7 days before semen sample collection. When at least three follicles were larger than 18 mm in diameter, human chorionic gonadotrophin (HCG, Pregnyl, Merck) was given at a dose of 5000–10 000 U, and ultrasound‐guided vaginal egg retrieval was performed 35–36 h later under intravenous anesthesia.

Oocytes were briefly rinsed in 80 IU/mL hyaluronidase (Hyase; Vitrolife), and cumulus cells were removed by repeated gentle aspiration. Denuded oocytes were washed and incubated in fresh G‐IVF medium. To ensure minimal manipulation exposure (<15 min per dish), each ICSI dish contained no more than four mature oocytes during manipulation. Spermatozoa with motility and normal head morphology were transferred into polyvinylpyrrolidone (PVP) buffer and then injected into the selected oocyte by using a microinjection needle. The subsequent culture of the injected oocytes was conducted in the G‐IVF medium using a humidified incubator with the following conditions: 37°C with the air condition of 6% CO_2_ and 5% O_2_, with 95% humidity.^15^

The formation of pronucleus (PN) was observed at 16–18 h after ICSI, and normal fertilization was confirmed with 2PN.

On Day 3 (66–68 h after fertilization), embryonic morphology was assessed according to the Istanbul consensus workshop on embryo assessment.^16^ The following conditions are defined as high‐quality embryos^15^: (1) normal fertilization, (2) at least six blastomeres, and (3) almost equal size of the blastomere, (4) percentage of embryo fragments not more than 20%, (5) transparent blastomere, without severe cytoplasmic inclusions or vacuoles, and (6) no multinucleate blastomere. On the third day, 1–2 high‐quality embryos were selected for transfer, and the remaining embryos were frozen. Embryos were also frozen if they are not suitable for transfer due to hyperstimulation. For patients who were not pregnant after the first transfer or who did not undergo transfer after oocyte extraction, frozen embryo transfer was performed in the natural cycle.

### 2.5 Clinical outcome and follow‐up

Blood samples were taken 12–14 days after transfer to detect HCG. Clinical pregnancy was confirmed by the presence of a gestational sac and fetal heartbeat at the 4‐week ultrasound after embryo transfer. Spontaneous miscarriage within 12 weeks of gestation, or embryo discontinuation, is known as early miscarriage. Ectopic pregnancy refers to the implantation of the gestational sac outside the uterine cavity.

### 2.6 Observation indicators

According to the International Glossary on Infertility and Fertility Care,^17^ the normal fertilization rate was defined as the number of oocytes containing two pronuclei (2PN) divided by the total number of mature oocytes (MII). The Day 3 high‐quality embryo rate is the number of Day 3 high‐quality embryos divided by the number of normal fertilized oocytes. The clinical pregnancy rate is defined as the number of clinical pregnancies per 100 embryo transfer cycles. Spontaneous miscarriage is defined as the spontaneous loss of an intrauterine pregnancy prior to 22 completed weeks of gestational age. The live birth delivery rate is defined as the number of deliveries that result in at least one live birth per 100 embryo transfer cycles.

### 2.7 Statistical analyses

Statistical analysis was performed using SPSS 26.0 software (IBM Corp, New York, NY, USA). Data are expressed as the mean and interquartile range (IQR) and percentage. The Mann–Whitney *U* test was used for continuous data, and the Chi‐square test or Fisher's exact test was used for counting data to assess differences between groups. A multivariable linear regression analysis was conducted to assess the ICSI clinical outcomes (the normal fertilization rate and high‐quality embryo rate) between the AZFc microdeletion group and the control group, with the normal fertilization rate and high‐quality embryo rate as continuous outcome variables. The analysis was adjusted for female age, sperm concentration, and the controlled ovarian stimulation protocol. Additionally, a multivariable logistic regression analysis was employed to compare the clinical pregnancy rate and the live birth rate between the AZFc microdeletion group and the control group. Clinical pregnancy and live birth were considered as binary outcome variables, and adjustments were made for female age, sperm concentration, controlled ovarian stimulation protocol, and the number of transferred embryos. *p* < 0.05 was considered significant.

The baseline between the AZFc microdeletion group and the control group was balanced by propensity score matching (PSM).^18^ Both demographic and clinical factors that may have confounded the outcomes were taken into account, including female age, male age, female body mass index (BMI) at the cycle start, number of oocytes retrieved, protocols of controlled ovarian stimulation, and sperm concentration. A 1:2 nearest neighbor caliper matching method was used to match the data between the AZFc microdeletion group and control group, and a caliper (0.03) of 0.2 of the standard deviation of the logit of the propensity score (0.15) was used. A standardized mean difference (SMD) of characteristics distribution of <0.2 was considered indicative of a negligible difference between groups in the mean or prevalence of a covariate.

## 3 RESULTS

### 3.1 Study population

In this study, six thousand eight hundred and twenty‐eight patients with a sperm concentration of ≤5 million/mL underwent a Y chromosome microdeletion assay, and four hundred and sixty‐four (6.8%) were found to have AZFc microdeletion. Of the four thousand one hundred and fifty‐six patients (60.9%) with a sperm concentration of 1–5 million/mL, only one hundred and ninety‐five (4.7%) were identified to have AZFc microdeletion; two hundred and sixty‐nine (10.1%) of the two thousand seven hundred and sixty‐two patients (39.1%) with a sperm concentration of <1 million/mL were found to have AZFc microdeletion. Finally, we recruited eighty‐five male infertile patients with severe oligozoospermia and AZFc microdeletion, and the control group was composed of one hundred and forty individuals with severe oligozoospermia but without AZFc microdeletion, with a 1:2 propensity score. In total, two hundred and twenty‐five oocyte retrieval cycles and two hundred and fourteen first transfer cycles were conducted. As shown in (Table 1), no statistically significant difference was observed between the two groups in terms of male age, female age, female AMH, female BMI, or sperm concentration.

**TABLE 1:** Study population.

	AZFc microdeletion *n* = 85	Non‐AZF deletion *n* = 140	*p*‐value
Female age (year)	28.3, 26–31	28.5, 27–31	0.69
Female BMI (kg/m^2^)	21.3, 19.6–22.9	21.6, 19.5–23.5	0.34
Female AMH (ng/mL)	5.6, 2.8–7.5	5.4, 2.8–7.2	0.83
Male age (year)	30.5, 28–33	30.7, 28–33	0.49
Sperm concentration (million/mL)	0.74, 0.1–1.5	0.84, 0.1–1.5	0.60

*Note*: Results are reported as median, IQR. Mann–Whitney *U* test was used.

Abbreviations: AMH, anti‐Müllerian hormone; AZF, azoospermia factor; BMI, body mass index.

### 3.2 ICSI clinical outcomes between the two groups

The multivariable linear regression analysis result indicated no statistically significant difference in normal fertilization rate (73% vs. 80%, *p* = 0.17) and high‐quality embryo rate (44% vs. 58%, *p* = 0.07) between the AZFc microdeletion group and the control group. Besides, another multivariable logistic regression analysis demonstrated no statistically significant difference in clinical pregnancy rate (65% vs. 70%, *p* = 0.44) and live birth rate (58% vs. 64%, *p* = 0.31) between the AZFc microdeletion group and the control group (Table 2).

**TABLE 2:** AZFc microdeletion may not affect the outcomes of ICSI.

	AZFc microdeletion *n* = 85	Non‐AZF deletion *n* = 140	*p*‐value	Adjusted β/OR (95% CI)	Adjusted *p*‐value
Normal fertilization (median, IQR) %	73% (60, 86)	80% (67, 88)	0.09	−0.04 (−0.09, 0.02)	0.17
High‐quality embryos (median, IQR) %	44% (22, 75)	58% (33, 86)	0.04	−0.09 (−0.19, 0.01)	0.07
Transferred cycles (*n*)	80	134			
Clinical pregnancy (%)	65% (52/80)	70% (94/134)	0.43	0.79 (0.44, 1.43)	0.44
Miscarriage (%)	12% (6/52)	7.4% (7/94)	0.55	1.71 (0.53, 5.5)	0.37
Live birth (%)	58% (46/80)	64% (86/134)	0.33	0.74 (0.42, 1.31)	0.31

*Note*: Results are reported as median, IQR. A multivariable linear regression analysis to assess the normal fertilization rate and high‐quality embryo rate between the AZFc microdeletion group and the control group. The analysis was adjusted for female age, sperm concentration, and the controlled ovarian stimulation protocol. In comparing the clinical pregnancy rate and live birth rate between the two groups, a multivariable logistic regression analysis was employed with adjustments for female age, sperm concentration, controlled ovarian stimulation protocol, and the number of transferred embryos.

### 3.3 ICSI clinical outcomes in patients with various sperm concentrations

To further investigate the effects of sperm concentration on the clinical outcomes of ICSI, we conducted subgroup analysis and divided the patients into two subcategories: the low sperm concentration subcategory (sperm concentration ≥1 million/mL) and the extremely low sperm concentration subcategory (sperm concentration <1 million/mL), as shown in Table 3. In the low sperm concentration subcategory, no significant difference was observed between the AZFc microdeletion group and the control group in terms of the normal fertilization rate, high‐quality embryo rate, clinical pregnancy rate, miscarriage rate, and live birth rate.

**TABLE 3:** ICSI outcomes in various sperm *concentration* patients.

	Concentration ≥1 million/mL					Concentration <1 million/mL				
	AZFc microdeletion *n* = 32	Non‐AZF deletion *N* = 59	*p*‐value	Adjusted β/OR (95% CI)	Adjusted *p*‐value	AZFc microdeletion *n* = 53	Non‐AZF deletion *N* = 81	*p*‐value	Adjusted β/OR (95% CI)	Adjusted *p*‐value
Normal fertilization (%)	77% (67, 93)	81% (67, 89)	0.71	0.02 (−0.06, 0.1)	0.63	71% (50, 82)	80% (67, 88)	0.02	−0.08 (−0.15, −0.01)	0.03
High‐quality embryos (%)	45% (0.22, 0.89)	59% (0.31, 0.86)	0.48	−0.02 (−0.18, 0.15)	0.85	44% (22, 67)	58% (40, 82)	0.04	−0.14 (−0.25, −0.02)	0.02
transferred cycles (*n*)	29	56				51	78			
Clinical pregnancy (%)	79% (23/29)	63% (35/56)	0.11	2.41 (0.81, 7.21)	0.11	57% (29/51)	76% (59/78)	0.02	0.41 (0.19, 0.89)	0.02
Miscarriage (%)	8.7% (2/23)	11% (4/35)	0.99	0.84 (0.12, 5.9)	0.85	14% (4/29)	5.1% (3/59)	0.21	2.86 (0.58, 14.03)	0.19
Live birth (%)	72% (21/29)	54% (30/56)	0.09	2.2 (0.8, 6.05)	0.12	49% (25/51)	72% (56/78)	0.01	0.37 (0.18, 0.78)	0.01

*Note*: Results are reported as median, IQR. A multivariable linear regression analysis to assess the normal fertilization rate and high‐quality embryo rate between the AZFc microdeletion group and the control group. The analysis was adjusted for female age, sperm concentration, and the controlled ovarian stimulation protocol. In comparing the clinical pregnancy rate and live birth rate between the two groups, a multivariable logistic regression analysis was employed with adjustments for female age, sperm concentration, controlled ovarian stimulation protocol, and the number of transferred embryos.

In the subcategory with extremely low sperm concentration, the fertilization rate (71% vs. 80%, *p* = 0.03), the high‐quality embryo rate (44% vs. 58%, *p* = 0.02), the clinical pregnancy rate (57% vs. 76%, *p* = 0.02), and the live birth rate (49% vs. 72%, *p* = 0.01) of the AZFc microdeletion group were significantly lower than those of the control group. The AZFc microdeletion group had a slightly higher miscarriage rate (14% vs. 5.1%, *p* = 0.19) than the control group, though the difference was not statistically significant.

### 3.4 ICSI clinical outcomes in AZFc microdeletion patients

We then evaluated the relationship between sperm concentration and the clinical outcomes of ICSI in the AZFc microdeletion group. As shown in Table 4, the fertilization rate (71% vs. 77.0%, *p* = 0.02), the clinical pregnancy rate (57% vs. 79%, *p* = 0.03), and the live birth rate (49% vs. 72%, *p* = 0.03) of the extremely low sperm concentration subcategory were lower than those of the low sperm concentration subcategory. The miscarriage rate of the extremely low sperm concentration subcategory was higher than that of the low sperm concentration subcategory, but the difference was not statistically significant.

**TABLE 4:** ICSI clinical outcomes in AZFc microdeletion patients.

	Concentration ≥1 million/mL *n* = 32	Concentration <1 million/mL *n* = 53	*p*‐value	Adjusted β/OR (95% CI)	Adjusted *p*‐value
Normal fertilization (median, IQR) %	77% (67, 93)	71% (50, 82)	0.02	0.11 (0.02, 0.19)	0.02
High‐quality embryos (median, IQR) %	45% (22, 89)	44% (22, 67)	0.52	0.12 (−0.05, 0.28)	0.17
Transferred cycles (*n*)	29	51			
Clinical pregnancy (%)	79% (23/29)	57% (29/51)	0.04	3.48 (1.14, 10.57)	0.03
Miscarriage (%)	8.7% (2/23)	14% (4/29)	0.68	0.63 (0.09, 4.22)	0.63
Live birth (%)	72% (21/29)	49% (25/51)	0.04	3.07 (1.1, 8.62)	0.03

*Note*: Results are reported as median, IQR. A multivariable linear regression analysis to assess the normal fertilization rate and high‐quality embryo rate in AZFc microdeletion patients. The analysis was adjusted for female age, sperm concentration, and the controlled ovarian stimulation protocol. In comparing the clinical pregnancy rate and live birth rate between the two groups, a multivariable logistic regression analysis was employed with adjustments for female age, sperm concentration, controlled ovarian stimulation protocol, and the number of transferred embryos.

## 4 DISCUSSION

AZF microdeletion is a significant contributor to severe oligozoospermia, with a prevalence of approximately 2%–5%.^2^ In contrast, the incidence of AZF microdeletion in the general population is rare, at only 0.025%.^13^ Male infertility guidelines issued by both ASRM and EAU recommend screening for AZF microdeletion when the sperm concentration falls below 5 million/mL.^3^, ^4^ Some researchers have also suggested that screening for AZF microdeletion should be conducted when sperm concentration is below 1 million/mL.^19^ Accurate quantification of spermatozoa at extremely low sperm concentrations is challenging due to various factors such as semen volume and technical differences.^8^ In our study, patients with sperm concentrations below 1 million/mL had a significantly higher incidence of AZFc microdeletion (10.1%) than those with sperm concentration between 1 and 5 million/mL (4.7%, *p* < 0.01). Therefore, to avoid missed diagnoses, we recommend that AZF microdeletion screening be conducted in Chinese male infertility populations when the sperm concentration is less than 5 million/mL.

Males affected by AZFc microdeletion usually have severe oligozoospermia or nonobstructive azoospermia, and the chances of achieving a natural pregnancy are extremely low.^2^ Nevertheless, ICSI technology provides them with an opportunity to achieve fertility.^11^ In this study, we attempted to eliminate female influences by excluding female factors such as uterine malformation, intrauterine inflammation, and diabetes history. Moreover, we selected men with severe oligozoospermia but no AZFc microdeletion as the control group, with a 1:2 ratio of propensity score matching for female age, BMI, AMH, male age, sperm concentration, and protocols of controlled ovarian stimulation. Our results revealed that when the number of transferred high‐quality embryos was equivalent, there was no statistically significant difference in pregnancy outcomes between the AZFc microdeletion group and the control group.

Some studies have reported a reduction in the sperm fertilization rate in individuals with AZFc microdeletion^9^, ^10^; however, other clinical outcomes of ICSI, such as the quality of embryos and implantation rate, remain unclear. In one study, no difference in the rate of high‐quality embryos was observed; the average sperm concentration in the AZFc microdeletion group was 1.10 million/mL, while the control group had an average sperm concentration of 10.55 million/mL.^9^ Another study found that the AZFc microdeletion group had a lower implantation rate, more cycles without transferable embryos, a lower clinical pregnancy rate, and a lower live birth rate than the control group. However, the study included not only patients with sperm concentrations of less than 1 million/mL but also nonobstructive azoospermia patients with testicular spermatozoa obtained through microsurgery.^12^ The divergence in results from different studies is likely due to the varying concentrations and sources of spermatozoa. To further assess the effects of sperm concentration on the fertilization rate and high‐quality embryo rate, we divided the two groups into two subcategories, the low‐concentration subcategory (sperm concentration ≥1 million/mL) and the extremely low‐concentration subcategory (sperm concentration <1 million/mL) and conducted a subgroup analysis. Our results have revealed that in the low‐concentration subcategory, AZFc microdeletion did not have an impact on the fertilization rate or high‐quality embryo rate, nor did it affect the clinical pregnancy rate, miscarriage rate, or live birth rate. However, in the extremely low‐concentration subcategory, the AZFc microdeletion group showed a significant decrease in fertilization rate and high‐quality embryo rate compared to the non‐AZFc microdeletion group, as well as a decrease in clinical pregnancy rate and live birth.

The reasons for the decline in fertilization rates, clinical pregnancy rates, and live birth rates remain unclear. *DAZ* (deleted in azoospermia) is an important gene in the AZFc region, encoding an RNA‐binding protein that is specifically expressed in early germ cells.^20^ The absence of *DAZ* can lead to a lag in zygotene during meiosis and a decrease in chromatin condensation, resulting in defects in spermatogenesis.^21^ It may also play important roles in early embryonic development.^22^ The *CDY* gene, another gene located in the AZFc region, is mainly expressed in the round sperm nucleus and is involved in the histone‐to‐protamine transition during sperm maturation.^23^ Changes in protamine ratio, protein hypophosphorylation/hyperphosphorylation, microRNA dysregulation, methylation differences, and multi‐omics molecular differences are possible mechanisms that explain the effects of AZFc on fertilization rate and embryonic development.^24^

According to our results, patients with AZFc microdeletion who have higher sperm concentrations are more likely to have successful clinical outcomes after assisted reproductive technology (ART) treatment. Some andrologists attempt to improve sperm quality by boosting endogenous testosterone production.^25^ It has been demonstrated that FSH treatment might be beneficial for people with nonobstructive azoospermia (NOA), as it can activate certain signaling pathways in gonadal target cells, thereby enhancing sperm count and motility.^26^ Spermatogenesis is strictly controlled by the hormone environment in the testes, where testosterone and estradiol play fundamental roles,^27^ and aromatase is closely associated with the production of testosterone and estradiol. It has been observed that decreasing aromatase activity can result in an increase in the rate of sperm recovery in NOA patients.^28^ Moreover, tamoxifen, an anti‐estrogen medication, can improve both the quantity and quality of sperm produced in the testes of NOA patients.^29^ In this study, no FSH, aromatase inhibitors, or estrogen receptor antagonists were administered to the patients; nevertheless, drug therapy may be an option for those with severe oligozoospermia prior to ICSI treatment.

In this study, we considered several potential confounding variables including age, AMH, and BMI for females, as well as age and sperm concentration for males. Additionally, we stratified sperm concentration. Our findings suggest that AZFc microdeletion has a detrimental effect on the success of ICSI procedures, especially in cases of extremely low sperm concentration. However, it is essential to acknowledge that this study did not incorporate whole‐exome sequencing or analysis of the gr/gr deletion; hence, the possibility of severe oligozoospermia resulting from genetic mutations or gr/gr deletion cannot be definitively ruled out. Furthermore, this study focused solely on the development of early embryos post‐ICSI, without considering the perinatal stage. Therefore, further investigation is needed to determine the potential impact of AZFc microdeletion on the prevalence of birth defects in offspring. Moreover, in the group with concentration more than 1 million/mL, subgroup with AZFc microdeletion suggests favorable results rather than that with Non‐AZF microdeletion; additionally, in the group with non‐AZFc microdeletion, concentration below 1 million/mL shows slightly better outcomes, although no statistically significant difference was observed. This finding is challenging to provide a definitive explanation for at this stage. It is important to underscore that this study was retrospective in nature and involved a limited patient cohort, highlighting the necessity for large‐scale prospective studies in the future.

It is possible for AZFc microdeletion to be inherited, and male offspring will consequently face infertility. In certain cases, the deletion area in offspring may expand, resulting in azoospermia^30^, ^31^ Furthermore, it is predicted that genes associated with gonadal tumors are located on the Y chromosome, and studies have shown that microdeletion of the Y chromosome can lead to increased risk of testicular cancer.^32^ Consequently, andrologists should recommend genetic counseling for AZFc microdeletion patients prior to ART treatment. After being fully informed, patients have the option to choose between ICSI and preimplantation genetic testing (PGT). Since only a few patients chose PGT, this study did not involve PGT data.

## 5 CONCLUSION

We investigated the outcomes of ICSI treatment for those with AZFc microdeletion by using propensity score matching. Our results showed that in the subgroup of those with extremely low concentrations of spermatozoa, AZFc microdeletion not only decreased the fertilization rate and high‐quality embryo rate but also the clinical pregnancy rate and live birth rate. Furthermore, investigation is necessary to identify the mechanism, but increasing quantity and quality of spermatozoa might be of benefit to those with AZFc microdeletion to achieve better assisted reproductive outcomes.

## FUNDING INFORMATION

This study was supported by grants from the National Natural Science Foundation of China (grant 81873478, to L.H.), Hunan Provincial Grant for Innovative Province Construction (2019SK4012), and Research Grant of CITIC‐Xiangya (YNXM‐202116).

## CONFLICT OF INTEREST STATEMENT

The authors declare no conflict of interest.

## Data Availability

The data that support the findings of this study are available from the corresponding author upon reasonable request.
